# Perspectives on Artificial Intelligence in Dermatology: An International Cross-Sectional Study

**DOI:** 10.3390/medicina62040759

**Published:** 2026-04-15

**Authors:** Emmanouil Karampinis, Christina-Marina Zoumpourli, Aimilios Lallas, Zoe Apalla, John Paoli, Bengü Nisa Akay, Cristian Navarette-Dechent, Behera Biswanath, Nkechi Enechukwu, Peter Chai, Jie Liu, Olga Toli, Christina Kontogianni, Dimitrios Sgouros, Alexander Katoulis, Christofer Tzermias, Paweł Pietkiewicz, Enzo Errichetti

**Affiliations:** 1 Second Dermatology Department, School of Health Sciences, Aristotle University of Thessaloniki, 54124 Thessaloniki, Greece; zoimd@yahoo.gr; 2 Department of Dermatology, Faculty of Medicine, School of Health Sciences, University General Hospital of Larissa, University of Thessaly, 38221 Volos, Greece; 3 1st Department of Dermatology and Venereology, “Andreas Sygros” Hospital, Medical School, National and Kapodistrian University of Athens, 16121 Athens, Greece; christinazoumpourli@gmail.com; 4 First Department of Dermatology, Aristotle University of Thessaloniki, 54124 Thessaloniki, Greece; emlallas@gmail.com; 5 Department of Dermatology and Venereology, Institute of Clinical Sciences, Sahlgrenska Academy, University of Gothenburg, SE-405 30 Gothenburg, Sweden; john.paoli@gu.se; 6 Department of Dermatology, Faculty of Medicine, Ankara University, 06560 Ankara, Turkey; nisaakay@gmail.com; 7 Department of Dermatology, Escuela de Medicina, Pontificia Universidad Católica de Chile, Santiago 340, Chile; ctnavarr@gmail.com; 8 Department of Dermatology and Venereology, All India Institute of Medical Sciences, Bhubaneswar 751019, India; biswanathbehera61@gmail.com; 9 Dermatology Unit, Department of Internal Medicine, Nnamdi Azikiwe University, Nnewi PMB 5025, Anambra State, Nigeria; nkechienechukwu@gmail.com; 10 Royal New Zealand College of General Practitioners, Wellington 6140, New Zealand; peter.chai@silverdalemedical.co.nz; 11 Peking Union Medical College Hospital, Beijing 100730, China; liujie04672@pumch.cn; 12 Department of Dermatology, Oncoderm Center One Day Clinic, 45332 Ioannina, Greece; olgatolimail@gmail.com; 13 Department of Internal Medicine, InnKlinikum Altötting, 84503 Altötting, Germany; 14 2nd Department of Dermatology and Venereology, “Attikon” General University Hospital, Medical School, National and Kapodistrian University of Athens, 12462 Athens, Greece; disgo79@gmail.com (D.S.); alexanderkatoulis@yahoo.co.uk (A.K.); 15 Department of Dermatology, IQ Intensive Care Skin Clinics, 11528 Athens, Greece; 16 Zwierzyniecka Medical Center, Zwierzyniecka, 60-814 Poznań, Poland; pietkiewicz.pp@gmail.com; 17 Department of Experimental and Clinical Medicine, Institute of Dermatology, University of Udine, 33100 Udine, Italy; enzoerri@yahoo.it

**Keywords:** artificial intelligence, dermatology, multi-country, chatbots, AI in medicine, perspectives

## Abstract

*Background and Objectives*: Artificial intelligence (AI) has transitioned to an integral part of dermatology in only few years, yet perceptions of its use vary widely, reflecting diverse hopes, concerns, and perceived clinical utility. *Materials and Methods*: In this study, 300 dermatologists from 13 countries, representing a range of experience levels and AI usage statuses, were surveyed regarding the characteristics and applications of AI in dermatology. *Results*: Among respondents, 61.33% reported having used AI tools in clinical practice. Adoption of AI was observed across all age groups, countries, and experience levels. Analysis of the types of AI tools used revealed a strong reliance on general-purpose large language models (LLMs), with chatbots being the most frequently cited category, utilized by 58.15% of users. Younger clinicians demonstrated a significant preference for chatbots (*p* < 0.05). Country-specific patterns in AI adoption were also noted. The most highly rated expected benefit of AI in dermatology was improved diagnostic accuracy, while the primary concern centered on regulatory and ethical limitations, suggesting that the “AI revolution” in dermatology is currently constrained less by technical barriers and more by regulation considerations. Use of consent forms when AI use takes place was more frequently reported as mandatory by dermatologists who had never used AI, reflecting heightened caution among non-users (*p* = 0.03). Additionally, 75% of respondents agreed that formal training in AI is necessary, highlighting a significant gap in traditional medical education regarding emerging technologies.

## 1. Introduction

Artificial intelligence (AI) has moved beyond a speculative future concept and has become an integral part of modern life and medicine, with dermatology being no exception. AI-based technologies and popular chatbots, such as ChatGPT and Gemini, offer powerful tools that aid or may surpass conventional approaches to clinical dermatology evaluation. AI is increasingly transforming dermatology by improving the diagnosis and management of a wide range of skin conditions, including skin cancer, esthetic dermatology, and hair disturbances. Trained AI models have the capacity to demonstrate diagnostic accuracy in skin cancer detection equal to that of experienced dermatologists, while machine learning models support the prediction of treatment outcomes, enabling a more personalized and effective patient care [1,2]. Dermatology practice has been significantly altered by the development of AI technologies, which now serve as new tools in the hands of dermatologists and substantially influence clinical decision-making. For example, one study reported that when AI suggested a malignant diagnosis for a lesion initially suspected to be benign, 76% of dermatologists indicated they would be more likely to perform a biopsy [3]. Conversely, when AI suggested a benign diagnosis for a lesion suspected to be malignant, 73% reported that this would not reduce their likelihood of performing a biopsy. These findings raise an important question whether AI can prompt dermatologists to question their own clinical judgment. While those findings are not uniform across all clinicians, as personal factors such as age and clinical experience play a role, additional extrinsic factors also influence how dermatologists interact with and respond to AI-supported decision-making [3]. Specifically, these include country-related exposure to AI technology (Section 1.1), stage of AI development (Section 1.2) and AI trends in the domain (Section 1.3).

### 1.1. Country-Related Factors That Affect Perspectives and Practices on AI Integration into Dermatology

Based on country-specific studies reflecting dermatologists’ attitude on AI technology and how it affects the specialty, country-related differences in dermatologists’ perceptions of AI do appear to reflect the broader state of AI adoption, readiness, and healthcare technology trends in each region. A variety of clinicians’ practices and professional culture within dermatology were studied across China, Argentina, Marocco, India, Poland, Saudi Arabia, and Australia (Table 1) [4,5,6,7,8,9,10]. In a recent study assessing the public’s awareness of AI in dermatology, highest interest was noted in Singapore, Ireland, Australia, Philippines, New Zealand, and the United Arab Emirates. High internet access and exposure and digital engagement make populations in these countries more likely to actively search for and engage with emerging digital health technologies, including AI in dermatology [11]. For example, Ireland reports internet usage rates near 99% of the population, well above the EU average [12]. A heatmap presented in previous study created by Google Trends demonstrated that digitally advanced countries (U.S., Europe, Australia, New Zealand, Singapore) showed high interest rates of AI in dermatology and equatorial regions (parts of Africa, South America, Southeast Asia) showed least [11].The structure of national healthcare systems likely shape clinicians’ views on AI, as the technology has the potential to address country-specific limitations or encounters significant adoption obstacles [13]. Additionally, advanced economies with greater healthcare investment capacity are anticipated to demonstrate higher AI market revenues while the general AI adoption in Latin America, including Argentina, is emerging but still nascent compared with advanced economies. Saudi Arabia is actively deploying AI in real healthcare settings, including analytics, personalized medicine, research, and administrative systems, with projects and studies demonstrating real-world AI impact on efficiency and patient flow [14]. Globally, market reports and figures for 2024, state that North America is the leading region with about USD 285 million in dermatology AI market revenue and that Europe is the second-largest region with USD 213 million in 2024, reflecting the interest of advanced economies in AI technology investments [15].

The availability of certain AI tools such as chatbots and AI-empowered Total Body Scanners also play a significant role in forming country or region-based practices of AI in dermatology. Deep learning systems such as DeepSeek, which is largely used in China, are being used to enhance diagnostic accuracy and streamline clinical operations in Chinese tertiary hospitals, with tasks ranging from AI-driven chronic diseases management to cost-performance optimization of certain operations [16]. A recent systematic review discusses how different regulatory pathways (such as, EU MDR vs. U.S. FDA vs. China’s regulatory system) affect the approval and deployment of AI medical devices, including those used in dermatology. This shows that regulatory environments vary significantly by region and directly influence whether advanced AI tools can be marketed and adopted locally [17].

Disease-specific AI tools also influence dermatologists’ exposure and perceptions of artificial intelligence. For instance, AI-based skin cancer detection systems are more likely to be adopted in countries such as Australia and New Zealand, where skin cancer incidence is high, than in many African countries where such conditions are comparatively rare [18]. Moreover, the perceived effectiveness and generalizability of AI tools can shape clinicians’ willingness to adopt them [19]. Systems trained predominantly on images of lighter skin types may perform less accurately on darker skin, where many dermatological conditions present with distinct clinical features [20]. This limitation can reduce confidence in AI technologies among dermatologists practicing in regions with predominantly Black or darker-skinned populations, thereby discouraging investment in advanced AI systems [21]. It is worth mentioning that language-specific differences in generative AI were also reported when chatbots answered questions on dermatology. Moreover, the lack of linguistic diversity in AI systems creates a significant barrier to adoption for smaller indigenous population [22].

### 1.2. Evolution of Perspectives on AI Integration into Dermatology

Analyzing the evolution of dermatologists’ perspectives in tandem with technological milestones provides comprehensive insight into how perceptions, acceptance, and clinical integration have changed over time. Along with AI-related major breakthroughs in image analysis, regulatory approvals (FDA approval of the first AI skin cancer diagnostic tool), or the increased popularity of chatbots’ use, dermatologists’ views may shift in response to increased evidence, experience, and trust. AI technological advances can influence clinical attitudes, training requirements, physicians’ concerns and practice patterns reflecting AI integrating dermatology practice, offering an important parameter when assessing this co-evolution (Table 2).

For example, the rate of the respondents believing that AI will not replace dermatologists in their routine work in the foreseeable future changed from 96.26% in 2020 [10], to 73.6% in 2023 [8]. From 2020 to 2023, AI systems have demonstrated capabilities that can rival expert-level performance in specific, well-defined tasks. This growing recognition explains this observed change in dermatologists’ perceptions over time, reflecting not fear of replacement, but an understanding that AI will play a significant and transformative role in dermatologic practice. Concerns regarding poor diagnostic accuracy among dermatologists decreased from 53% of respondents in 2021 to 44.2% in [9], while importance of incorporating AI-empowered dermatology into medical education increased, with support rising from 79.8% in 2020 to 84.9% in 2023 [23].

**Table 2:** Key developments in dermatology and AI over time.

Time Period	Important Event
Before 2017	Dermatology processing techniques were evaluated by multiple studies mainly by computer science experts
2017	Esteva et al. introduced the concept of CNN in dermatology. The ISIC 2017 dataset was created to advance automated melanoma detection and improve machine learning models for dermoscopic image analysis [24].
2018	The HAM10000 dataset (“Human Against Machine with 10 000 training images”) was created and made publicly available as a large, multi-source collection of dermatoscopy images for research on automated skin lesion analysis [25].
2018	The first studies on AI and Cosmetic Dermatology are published [26]
2020	The collaboration of AI and dermatologist on the hotspot [27]. AI-assisted differential diagnosis of skin diseases was studied [28]
2021	Introduction of explainable AI in dermatology [29]. The need for diverse skin clinical images is acknowledged [30].
2022	Beginning of Generative AI with ChatGPT release.
2023	The first studies on dermatology-related ChatGPT-assisted assessment. Release of generic chatbots, including Gemini.
2023	In certain countries, AI applications were introduced in healthcare [31].
2024	FDA Approves First AI-Powered Skin Cancer Diagnostic Tool.
2025	New, more efficient chatbot versions appear. The need for standardization for chatbot-focused studies was identified.
2025–2026	The first books on Artificial intelligence and Dermatology were published as Applications of Artificial Intelligence on Dermatology: Dermatology Ex Machina Clinical applications [32]. First congress on the Artificial Intelligence and Dermatology will be held in Vienna in September in 2026 [33].

### 1.3. Study Trends That Affect Perspectives on AI Integration into Dermatology—Bibliometric Analysis on Dermatology and Artificial Intelligence

Research trends in AI and Dermatology can both guide and limit dermatologists’ perspectives as high-performance studies increase optimism for specific clinical applications while those focusing on the explainability and bias analyses of the AI models may create cautious trust. The 15 most cited studies in this field were selected, while notes and conference papers were excluded (Table 3). Our review included studies focusing on advanced AI methodologies, such as the development and application of CNNs and related techniques. The most studies detected included close collaboration between dermatologists and AI engineers, highlighting the importance of interdisciplinary cooperation. Indeed, although AI engineers have strong expertise in algorithm design, model optimization, data processing, and advanced AI techniques, the clinical relevance and the accurate disease images labeling secured by expert dermatologists, making them indispensable for defining clinically meaningful AI research. The most cited research items are predominantly centered on AI-assisted technologies for skin cancer detection, as well as on methodological improvements (CNN fusion-based methods, segmentation processes, etc.) aimed at enhancing AI performance in this area [24].

Collectively, highly cited studies demonstrating AI models equal performance or even superiority may create a division in dermatologist perspectives: admiration for AI’s capabilities and respect and optimism for AI’s potential or concern for overreliance and risks of skilling degradation. Across the spectrum, maintaining clinical oversight and professional responsibility remains essential and the AI- dermatologist collaboration, which has a central role, is reported more and more in recent publications on the topic. Worth mentioning is that computer engineering techniques and concepts are referenced frequently, which creates the need for dermatologists to have a deeper understanding of these aspects. Therefore, appropriate training in this area should be considered a necessary component of dermatology practice. Indeed, in the most recent study assessing dermatologist beliefs on AI, only 24.6% reported good or excellent knowledge, indicating a substantial gap in advanced understanding [9].

## 2. Materials and Methods

A cross-sectional survey was conducted among dermatology fellows between 10 January 2025, and 10 January 2026. A structured, self-administered questionnaire was distributed electronically using Google Forms. The questionnaire was validated prior to distribution to ensure its reliability and relevance. Content and face validity were established through review by experts in dermatology and digital health, confirming that the questions appropriately covered key aspects of AI integration. The survey achieved a response rate of 68%, while it was distributed via email invitations, which included a brief explanation of the study and a link to complete the survey online. Invitations were sent specifically to practicing dermatologists, ensuring that the responses accurately represented the perspectives and experiences of professionals within the field. Participation was voluntary and anonymous. The study focused on the above-mentioned countries because the authors had direct access to dermatologist colleagues in these regions, which facilitated effective survey distribution and ensured a higher response rate. The questionnaire collected demographic and professional information, including age bin, years of experience in dermatology, predominant country of practice, and use of AI in clinical practice. Participants were also asked to specify the types of AI tools they routinely use and their primary clinical applications. Perceptions of AI were assessed using a five-point Likert scale. Respondents evaluated current AI capabilities in dermatology, including its role in education, diagnostic accuracy, workload reduction, and treatment monitoring. Additional items explored expectations regarding future AI developments, such as its potential use in underserved areas and the anticipated degree of integration into clinical workflows. Concerns related to AI implementation were also assessed, including reliability, medico-legal issues, ethical considerations, and the lack of regulatory frameworks governing AI use in dermatology. Continuous variables were summarized using means and standard deviations, while categorical variables were reported as frequencies and percentages. Categorical variables were analyzed using the chi-square test. Numerical variables were first assessed for normality using the Shapiro–Wilk test. For variables demonstrating non-normal distributions, comparisons were performed using the Kruskal–Wallis test, whereas one-way ANOVA was applied to normally distributed data. A *p*-value of <0.05 was considered statistically significant.

## 3. Results

### 3.1. Demographics

A total of 300 dermatology fellows participated in the survey. The majority of respondents were aged 30–39 years (40.45%), followed by those under 30 years (30.91%). Female participants accounted for 62.0% of the sample. When stratified by continent, nearly a third of the respondents practiced in Asia (31.0%), followed by Europe (29.3%), Africa (18.7%), South America (14.3%), and Oceania (6.7%). Regarding professional experience, most participants had less than 5 years of dermatology practice (36.0%), followed by 5–10 years (26.3%), 11–20 years (21.0%), and more than 20 years (16.7%) (Table 4).

**Table 4:** Demographic data of the survey participants (n = 300).

Category	n	%n
Age (years)		
<30	68	30.91%
30–40	89	40.45%
40–49	37	16.82%
50–59	18	8.18%
60+	8	3.64%
Sex		
F	186	62%
M	114	38%
Country		
Turkey	49	16.33%
Greece	28	9.33%
Sweden	24	8%
India	27	9%
China	17	5.67%
Chile	43	14.33%
UK	22	7.67%
New Zealand	20	6.67%
Germany	13	4.33%
Nigeria	16	5.33%
Burkina Faso	17	5.67%
Mali	14	4.67%
Cote D’ Ivoire	9	3%
Grouping by continent		
Europe	88	29.33%
Africa	56	18.67%
Asia	93	31%
South America	43	14.33%
Oceania	20	6.67%
Experience (years)		
<5	108	36%
5–10	79	26.3%
10–20	63	21%
>20	50	16.7%

### 3.2. Use of AI

In total, 61.33% of dermatology fellows reported having used AI tools in clinical practice. AI adoption was distributed evenly across all age (*p* = 0.90) and experience (*p* = 0.56) groups. While the raw percentage of male users (65.79%) was higher than that of female users (58.60%), females represented a larger share of the total “Yes” responses (59.24%) due to their higher overall representation in the study but without statistical significance (*p* = 0.263). Across countries, Germany, Greece, and Sweden (>75%) show the highest rates of reported AI usage (while the lowest adoption was observed in Nigeria (31.25%) and Turkey (51.02%). Most countries hover between the 50% and 70% mark, suggesting a relatively widespread (though varied) use of AI tools globally within the surveyed group. However, geographic location was not a primary determinant of AI adoption within the study population (chi-square *p* = 0.247). The restricted number of participants representing some countries needs to be acknowledged.

The frequency of reported AI tools indicates a strong reliance on general-purpose Large Language Models (LLMs). Chatbots were the most frequently cited category, utilized by 58.15% of the user cohort (n = 107). Mobile applications represented the second most common toolset at 33.70% (n = 62), while specialized clinical systems, including lesion classification software and camera-based imaging tools, were utilized by 19.57% of the participants (n = 36). While overall AI adoption was consistent across age groups, a Chi-square test revealed a significant association between age and the specific type of AI tool utilized, (*p* = 0.003). Younger participants (<30 years old) showed a strong preference for chatbots (73.3%), whereas participants in the 50–59 age group were more likely to utilize specialized clinical and lesion classification systems (40.0%) compared to their younger counterparts. That is more likely due to the fact that older dermatologists have the ability to afford such systems. Additionally, a highly significant association between the country of practice and the specific type of AI tool utilized by healthcare professionals was observed (*p* < 0.001). Qualitative analysis of the data revealed distinct regional preferences: while practitioners in China (100%,) and Turkey (79%), predominantly utilized chatbots, those in African countries and New Zealand (50%) showed a higher frequency of mobile application usage. We have noticed high positive correlation between professional experience and AI tool type (*p* < 0.001). Similar to the age variable, the data shows that “seniority” in the medical field drastically changes how a professional interacts with AI. Junior professionals were overwhelmingly reliant on Chatbots (62.9% of their tools). This suggests a reliance on LLMs for quick information retrieval or drafting during the early stages of practice while professionals with over 20 years of practice are the only group where advanced clinical systems (41.0%) are the most frequently used tool, outperforming Chatbots (23.1%).

The selection of AI technology was significantly influenced by clinical focus (*p* = 0.001). Specialized clinical systems and mobile applications were most frequently utilized for dermato-oncology, whereas chatbots were predominantly used for general dermatology and hair disorders (77.0% and 67.5%, respectively). This suggests that clinicians prioritize visual-recognition AI for oncological diagnostics while favoring generative language models for chronic inflammatory and hair disorders. There was a positive association between the dermatology area and scope of use (*p* = 0.001). In dermato-oncology, AI tools are overwhelmingly used for diagnostic purposes (50.7%). Responses focused on general dermatology were more evenly split between diagnostic use (36.9%) and treatment guidance (35.2%). A highly significant association between the type of AI tool employed and its clinical scope of practice was reported (*p* < 0.001). Chatbots demonstrated the broadest clinical utility, serving as the primary tool for treatment planning, as LLMs are text-based, they are uniquely suited for “reasoning” tasks like drafting treatment plans, explaining drug interactions, and providing patient education. For mobile apps, diagnosis is the primary driver (56% of their use) while clinical systems (like FotoFinder) are used almost as much for follow-up as they are for diagnosis.

### 3.3. Hopes and Concerns Related to AI Use in Dermatology

Participants rated diagnostic accuracy as the greatest benefit of AI tools, whereas treatment monitoring received the lowest ratings (Table 5). Perceptions of workload reduction and educational utility of AI were similar. Regarding AI-related concerns, the primary obstacle identified by dermatologists was the lack of regulations, closely followed by ethical concerns and vague legal framework. Interestingly, the safety of predictions scored lowest among the four categories. These results suggest that for the medical dermatology community, the ‘AI Revolution’ in dermatology is currently stalled not by technical limitations, but by regulatory and ethical barriers. However, statistically significant differences were not reported amongst the groups.

**Table 5:** Evaluation of AI Dermatology Applications: Mean Participant Ratings and Standard Deviation.

	Mean Likert Score, SD
Current capacities	
AI can improve diagnostic accuracy in dermatology and specifically in skin cancer detection	3.78 ± 0.83
AI can reduce the workload of dermatologists.	3.56 ± 0.97
AI can better monitor treatment outcomes.	3.33 ± 1.00
AI can educate new dermatologists	3.56 ± 1.11
Future perspectives	
AI tools will become an integral and indispensable part of clinical practice in the nearest future	3.86 ± 0.96
AI will become an important dermatology tool in underserved areas.	3.81 ± 1.06
Concerns	
Reliability of AI predictions	3.36 ± 1.09
Vague Legal frameworks in the use of AI	3.54 ± 1.10
Ethical issues (bias, patient data privacy and clinician-patient relation disturbance)	3.58 ± 1.12
Lack of regulation and standardization	3.66 ± 1.08

Country-correlated responses using Kruskal–Wallis test indicated no significant differences between countries in agreement all the statements, future perspectives and concerns (*p* > 0.05). Regarding differences amongst AI tool users and non-users, educational use of AI is the only category where experience in using AI significantly changed professional opinion (*p* = 0.049). Dermatologists who used AI tools are significantly more confident in the technology’s ability to train and mentor the next generation of doctors. Regarding the impact of age dermatologists below 40 years of age felt more experienced with digital technology, yet significantly more skeptical about AI’s reliability than their older colleagues (*p* < 0.001).

### 3.4. Consent and Training on AI-Empowered Dermatology

Nearly 60% of participants believe that patient consent should be mandatory for using AI tools. This highlights a strong professional consensus on the importance of patient autonomy and informed choice. The high “Yes” and “Maybe” total (approx. 88%) reinforces the previous findings regarding ethical issues and vague Legal Frameworks, which achieved the highest scores in the concern section. Therefore, dermatologists-responders lean toward mandatory consent as a way to protect both the patient and the practitioner.

Regarding a country-specific approach, clinicians form India, Mali, and Burkina Faso demonstrate the strongest support for mandatory consent, with no respondents opposing its requirement. However, the small number of participants from certain countries prevented us from drawing definitive conclusions.

Lastly, regarding training, over 75% of respondents agree that formal training in AI is necessary, recognizing a significant gap in their traditional medical education regarding emerging technologies. Whether the dermatologist replied is an early adopter (“Used AI”) or a skeptic (“Never Used AI”), exactly 75% of both groups agree that formal training should be part of medical school or residency. Additionally, there is no significant difference between countries regarding the desire for training. Whether in Europe, Asia, Africa, or North America, most doctors agree that AI training should be integrated into the medical curriculum (*p* = 0.43).

## 4. Discussion

Dermatologist participants in our study reflect contemporary perspectives within the dermatology community regarding AI integration and its impact on clinical practice during the 2025–2026 period. This timeframe is particularly relevant given the rapid evolution of AI technologies, including increasingly sophisticated large language models, advanced imaging systems embedded in cameras and diagnostic tools that rely on convolutional neural networks trained on billions of images. A growing trend toward AI acceptance in dermatology is evident when comparing our findings with previous studies. In 2020, approximately 85% of dermatologists reported never using AI in clinical practice, with only 15% reporting active use [46]. By 2025, this proportion had shifted, with 56.6% reporting no clinical AI use, implying that 43.4% had adopted AI tools [9]. In our study group, 61.33% of participants reported using AI in clinical practice, suggesting a continued increase in AI adoption over time.

Contrary to findings reported in prior work [47], we observed that younger age was associated with greater skepticism toward AI use. Although younger dermatologists tend to be more technologically proficient, they may also be more aware of current AI limitations, including algorithmic bias, overfitting, and limited generalizability across diverse patient populations. Early-career dermatologists are often trained to critically evaluate emerging technologies, which may lead them to question the robustness, validation, and real-world clinical applicability of AI tools before fully integrating them into decision-making processes.

Educational applications of AI emerged as the most debated topic in our cohort, although support was strongest among dermatologists with prior AI experience, who viewed AI as a potentially valuable educational tool. While previous studies suggest that AI-generated cases can offer useful educational insights, concerns remain that such cases may lack real-world clinical reality and could mislead users if not carefully validated. Although the potential of AI to support underserved areas was rated highly, an important question remains regarding whether these AI models will be sufficiently available and accessible to the populations and healthcare providers who need them most, and whether the necessary infrastructure and knowledge will be in place to enable their effective use in resource-limited settings [48].

Furthermore, dermatologists using AI tools viewed patient consent as less essential than those who did not use such tools. Practical experience with AI may normalize its role as a background clinical support tool rather than a distinct intervention, particularly when AI functions without direct patient interaction. Conversely, non-users may view AI as a novel or opaque technology, increasing ethical concerns related to autonomy and transparency. Country-based differences in attitudes toward consent forms were also observed, likely reflecting variations in legal frameworks, cultural norms, healthcare systems, and levels of digital health integration, as discussed in Section 1.1.

Notably, training in AI was almost universally requested, with exceptionally high levels of agreement across respondents. This consensus likely reflects widespread recognition that AI is increasingly present in clinical practice while formal training remains insufficient. Dermatologists appear to agree that education is essential to ensure safe interpretation of outputs and to control risks such as bias or overreliance [49].

Also, we identified correlations between AI use and specific tools, clinical scopes, and dermatologic entities. Large language models, such as ChatGPT and Gemini, were the most commonly used AI tools. Skin cancer and its mimickers represented the most frequently cited clinical applications, with many dermatologists using advanced imaging systems such as total body photography scanners and, in some cases, uploading images to AI-based platforms. A clear association was observed between skin cancer management and camera-based AI systems, while inflammatory dermatoses were more commonly addressed using chatbots. Chatbots demonstrated the broadest clinical utility, particularly for treatment planning, as their text-based nature makes them well suited for reasoning tasks, including drafting management plans, explaining drug interactions, and supporting patient education [50,51]. However, the risks of chatbot overreliance need to be outlined. Overreliance on chatbots may reduce diagnostic accuracy because they cannot replace full patient assessment. Excessive use may increase the risk of misdiagnosis, delayed treatment, and inappropriate clinical decisions [52]. As AI technologies continue to evolve, the clinical correlations of the tools may change, and more options may appear. For example, the introduction of AI-guided systems in laser therapies to improve precision, safety, and clinical outcomes, would encourage dermatologists to increasingly incorporate AI into treatment practices [52].

Finally, the highest score regarding future directions and hopes was reading the diagnostic accuracy improvement of cutaneous lesions as skin cancer and the greatest concern is the lack of regulation. While dermatologists may trust the technical promise of AI, the absence of clear regulatory frameworks raises fears of misuse, variability in standards, and medico-legal consequences. Together, these findings highlight a gap between technological optimism and systemic readiness, indicating the need for regulation to ensure that gains in accuracy are translated into safe, ethical, and standardized clinical practice. Those findings indicate that AI is moving rapidly to practical adoption in dermatology practice, but trust, regulation, and education remain key factors influencing its integration and worry clinicians. Chatbots lead adoption because of convenience, but standardized guidelines or specifically trained dermatology chatbots are needed to ensure safe and effective clinical use.

This study has several limitations that should be acknowledged. First, European-based respondents may have a stronger focus on skin cancer compared with dermatologists from Africa, Asia, and South America, reflecting regional differences in disease prevalence and access to specialized equipment due to economic factors. Second, selection bias may have occurred, as the authors distributing the questionnaire have a background in skin cancer, which could have influenced the responses toward oncology-related applications. Third, the number of participants from certain countries was limited, reducing the generalizability of findings for those regions. Finally, many of the advanced AI tools currently available focus on skin cancer, with limited applicability to inflammatory or non-oncologic dermatologic diseases, which may have influenced respondents’ preferences and perceptions of AI integration.

## 5. Conclusions

Perspectives from dermatologists in 2026 indicate a higher rate of AI adoption to clinical reality compared with earlier studies, reflecting the rapid advancement of sophisticated AI models and the introduction of new camera-based systems that have enhanced clinical performance. Educational applications of AI in dermatology gathered the most heterogeneous responses, with younger dermatologists expressing greater skepticism toward AI use. Large language model-based chatbots emerged as the most commonly used AI tools, likely due to their widespread accessibility and ease of use. Despite optimism regarding AI’s clinical potential, the most prominent concern reported was the lack of clear regulatory frameworks governing its use. Notably, no significant country-based differences were observed in all categories, statements, hopes and concerns related to AI, suggesting a broadly shared global perspective on both the opportunities and challenges of AI integration in dermatology.

## Figures and Tables

**Table 1:** A list of regional factors shaping how AI is perceived and applied in dermatology.

**Regional Factors That Affect Perspectives and Practices on AI Integration into** **Dermatology**
1. Technology Lifestyle and Digital Readiness 2. Healthcare System Structure and Dermatology Practice Model 3. Economic Development and AI Technology Investment to Healthcare 4. Regulatory Environment 5. Region-specific Availability of AI Tools 6. Disease Epidemiology and Clinical Demand of specific AI tools 7. AI Dataset Diversity and Population of Interest 8. Exposure to AI during dermatology education system

**Table 3:** A Summary of Core Outcomes and Evidence from Landmark Studies in AI-Supported Dermatology.

Study	Citations (Until 18 January 2026)	Core Message
[24]	10,851	A specific-trained CNN based on 129,450 clinical images can achieve performance on par with all tested expert, showing that an artificial intelligence model can be capable of classifying skin cancer with a level of competence comparable to dermatologists. The study includes the hallmark of Dermatology and AI research.
[34]	1009	CNNs with more than 50 layers, can significantly improve automated melanoma recognition from dermoscopy images compared with earlier, shallower methods.
[27]	702	Good quality AI-based support of clinical decision-making improves diagnostic accuracy over that of either AI or physicians alone, and that the least experienced clinicians gain the most from AI-based support.
[35]	652	Evaluation of a deep learning-based framework for classifying skin diseases using a combination of MobileNet V2, a CNN architecture, and Long Short-Term Memory units to improve classification accuracy and maintain contextual feature information.
[28]	621	Presentation of a deep learning system to provide a differential diagnosis of skin conditions using 16,114 de-identified cases (photographs and clinical data).
[36]	594	Performance of a deep learning algorithm to classify the clinical images of 12 skin diseases was compatible with dermatologist performances.
[37]	541	High accuracy rates of melanoma scored by an AI framework based on the ISIC 2017 dataset evaluation.
[38]	526	Deep learning with established machine learning approaches, an AI system capable of segmenting skin lesions and analyzing the detected area and surrounding tissue for melanoma detection achieved high accuracy than dermatologists.
[39]	465	A detailed systematic review of deep learning techniques for the early detection of skin cancer.
[40]	432	A CNN trained by open-source images exclusively outperformed 136 out of 157 dermatologists, confirming the potential of AI to serve as a highly effective clinical decision-support tool in dermatology.
[41]	427	Development of multitask CNN, trained on multimodal data (clinical and dermoscopic images, and patient metadata) can classify the 7-point melanoma checklist criteria and perform skin lesion diagnosis.
[42]	423	Machine-learning classifiers outperformed human experts in the diagnosis of pigmented skin lesions, and they should be part of clinical workflow and practice.
[43]	421	A novel deep learning segmentation methodology of skin lesions was proposed, with full spatial resolutions of the input image enabling the AI model to learn better specific and prominent features.
[44]	385	A systemic review on CNNs that display a high performance as skin lesion classifiers
[45]	353	Ensemble framework of CNNs were proposed for skin lesion classification, based on different fusion-based methods

## Data Availability

The data described in this study are available upon request from the corresponding author.
